# Using rodent data to elucidate dopaminergic mechanisms of ADHD: Implications for human personality

**DOI:** 10.1017/pen.2023.12

**Published:** 2024-01-31

**Authors:** Gail Tripp, Jeff Wickens

**Affiliations:** 1 Human Developmental Neurobiology Unit, Okinawa Institute of Science and Technology Graduate University, Okinawa, Japan; 2 Neurobiology Research Unit, Okinawa Institute of Science and Technology Graduate University, Okinawa, Japan

**Keywords:** Dopamine, reward, ADHD

## Abstract

An altered behavioral response to positive reinforcement has been proposed to be a core deficit in attention deficit hyperactivity disorder (ADHD). The spontaneously hypertensive rat (SHR), a congenic animal strain, displays a similarly altered response to reinforcement. The presence of this genetically determined phenotype in a rodent model allows experimental investigation of underlying neural mechanisms. Behaviorally, the SHR displays increased preference for immediate reinforcement, increased sensitivity to individual instances of reinforcement relative to integrated reinforcement history, and a steeper delay of reinforcement gradient compared to other rat strains. The SHR also shows less development of incentive to approach sensory stimuli, or cues, that predict reward after repeated cue-reward pairing. We consider the underlying neural mechanisms for these characteristics. It is well known that midbrain dopamine neurons are initially activated by unexpected reward and gradually transfer their responses to reward-predicting cues. This finding has inspired the dopamine transfer deficit (DTD) hypothesis, which predicts certain behavioral effects that would arise from a deficient transfer of dopamine responses from actual rewards to reward-predicting cues. We argue that the DTD predicts the altered responses to reinforcement seen in the SHR and individuals with ADHD. These altered responses to reinforcement in turn predict core symptoms of ADHD. We also suggest that variations in the degree of dopamine transfer may underlie variations in personality dimensions related to altered reinforcement sensitivity. In doing so, we highlight the value of rodent models to the study of human personality.

Attention deficit hyperactivity disorder (ADHD) is a prevalent disorder defined by persistent and developmentally inappropriate levels of inattention, and/or hyperactivity and impulsivity. Although generally considered a neurodevelopmental disorder with a neurobiological and genetic basis, the pathophysiology of ADHD is unknown. According to the fifth edition of the Diagnostic and Statistical Manual (DSM-V) diagnosis is based on the presence of a number of reported behavioral symptoms, in some combination of inattention, hyperactivity, and impulsivity, together with functional impairment (Stein, Lund, & Nesse, 2013). The International Classification of Diseases-11 (ICD-11) uses the same symptoms to define ADHD with the addition of an impulsive trait description that in DSM-5 is represented by specific symptoms (Gomez, Chen, & Houghton, 2023).

Like many psychiatric disorders, ADHD is thought to be the product of multiple interacting causes acting on multiple brain mechanisms. The DSM-V and ICD-11 criteria provide a reliable communication system providing reference categories for biomedical and psychological research. However, these classification systems are not meant to imply that disorders like ADHD are discrete with specific causes and biomarkers and distinct boundaries (Stein et al., 2013). All the symptoms of ADHD occur in typically developing individuals to some extent. For example, from the DSM-5 one can select symptoms like: “Is often forgetful in daily activities”; “Often loses things necessary for tasks and activities”; “Often fails to give close attention to details”; “Often talks excessively”; and “Often interrupts or intrudes on others (e.g., butts into conversations or games).” In isolation, such behaviors are not uncommon in the wider population. Empirically, ADHD-like symptoms are distributed throughout the population on a continuum (Arcos-Burgos & Acosta, 2007). The differences between individuals with ADHD and typically developing individuals are not qualitative but quantitative.

The continuum of symptoms raises the possibility that ADHD could be viewed as an exaggeration of normal personality traits. It may be more accurate to consider ADHD as a continuous phenotype rather than a categorical “with” or “without” ADHD dichotomy (Levy, Hay, McStephen, Wood, & Waldman, 1997). A dimensional model (Hierarchical Taxonomy of Psychopathology, HiToP) has been proposed, which includes ADHD, and unlike the traditional classification systems typified by DSM-5 and ICD-11, uses dimensions instead of symptom clusters.

To date, however, ADHD has not been consistently located within the HiToP model (Mullins-Sweatt et al., 2022). For example, within HiToP, ADHD is included under the antisocial subfactor, which combines disinhibited externalizing and antagonistic externalizing spectra, along with antisocial personality disorder, oppositional defiant disorder, conduct disorder, and intermittent explosive disorder (Mullins-Sweatt et al., 2022). In contrast to the symptoms of antisocial personality disorder (such as acting recklessly, breaking laws without caring about consequences, and disregarding responsibilities), the ADHD symptoms listed in DSM-5 and ICD-11 definitions do not necessarily cause conflict with others in the same way (De Pauw & Mervielde, 2011). Although individuals with ADHD may have difficulty following the rules, it is not for want of trying. In addition, ADHD has been found to be a relatively weak indicator of externalizing factors (Carragher et al., 2014) and to also involve internalizing spectra (Bozhilova, Michelini, Kuntsi, & Asherson, 2018; Nigg, Karalunas, Feczko, & Fair, 2020). Thus, ADHD may not be well placed under the antisocial subfactor in HiToP. Further work is needed to develop an appropriate dimensional concept for ADHD.

In an ideal framework for conceptualizing ADHD, dimensions would be based on neurobiological causes. This is beyond the present state of the art in neurobiological research. However, there are many results of neurobiological research that are highly relevant to understanding the pathophysiology of ADHD and it is useful to include them in theoretical approaches and emerging dimensional frameworks. For example, we and others have suggested that many of the symptoms of ADHD arise from an altered sensitivity to reinforcement (Catania, 2005; Iaboni, Douglas, & Baker, 1995; Sagvolden, Aase, Zeiner, & Berger, 1998; Tripp & Wickens, 2008, 2009; Wickens & Tripp, 1998; Williams & Dayan, 2005).

Central to our thesis is the role of dopamine in positive reinforcement, and by implication, in altered reinforcement sensitivity in ADHD and related personality traits. Although widely regarded as a “reward molecule” in the popular literature, the behavioral and physiological effects of dopamine are complex and deeply involved in multiple fundamental aspects of brain function (Wise, 2004). Here we focus on the implications of two aspects of dopamine function: the firing patterns of dopamine neurons in response to reward and reward-predicting cues (Schultz, 2002), and the timing-sensitive effects of dopamine on the strength of synaptic connections at the cellular level (Reynolds, Hyland, & Wickens, 2001; Shindou, Shindou, Watanabe, & Wickens, 2019). While these findings from animal studies may seem remote from human psychopathology and personality theories, behavioral characteristics are profoundly affected by subtle alterations in these mechanisms. We review dopamine dynamics in positive reinforcement in section 1. We then describe the behavioral characteristics of a rat with altered sensitivity to reinforcement in section 2. Finally, we consider the implications of altered behavioral characteristics for healthy humans and individuals with ADHD in section 3.

## 1. Dopamine dynamics and timing in positive reinforcement

### 1.1 Transfer of the dopamine response from reward to reward-predicting cues

**Figure 1.**
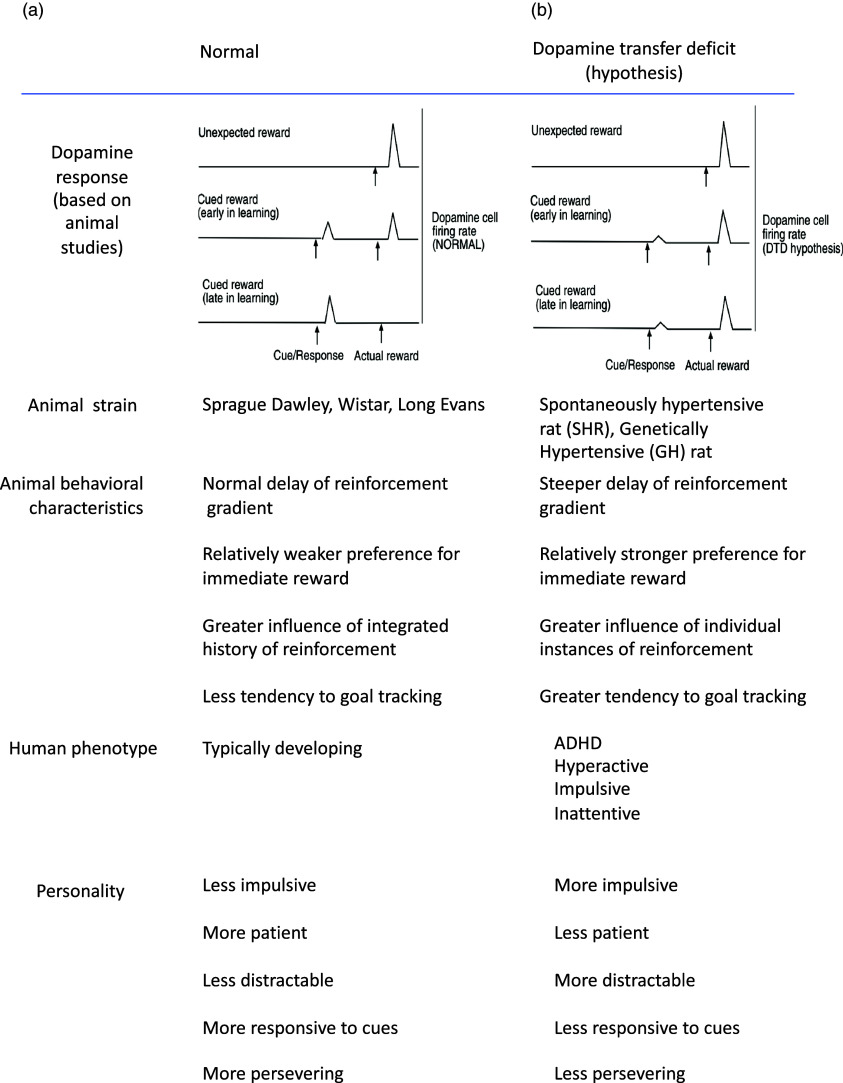
Transfer of dopamine response from actual reward to cues predicts behavioral characteristics. Traces show idealized dopamine signal in normal (a) and hypothesized dopamine transfer deficit (b). In both cases, unexpected primary reward causes a dopamine response. Normally, after repeated pairing of cue and reward, the dopamine response transfers to the cue. When there is a dopamine transfer deficit the cue response fails to develop as strongly as normal, and the response to the actual reward persists. Compared to normal rat strains, the SHR shows a dopamine transfer deficit. This is associated with characteristic of immediate over-delayed reward. In humans, a dopamine transfer deficit may give rise to symptoms of ADHD. These can be viewed as extremes of normal variations in individual personality traits.

Extensive human and animal research over the past few decades has revealed a central role for dopamine in positive reinforcement. In non-human primates and rodents, experiments have shown that dopamine neurons in the midbrain are activated by unexpected primary rewards, such as sips of juice or pieces of apple delivered at random times (Ljungberg, Apicella, & Schultz, 1992; Schultz, Apicella, & Ljungberg, 1993; Schultz, Dayan, & Montague, 1997). However, when these primary rewards are repeatedly preceded by a sensory stimulus (such as a sound tone or light), the response of the dopamine neurons to the reward decreases, and the response to the associated sensory stimuli increases (Day, Roitman, Wightman, & Carelli, 2007; Ljungberg et al., 1992; Pan, Schmidt, Wickens, & Hyland, 2005; Schultz et al., 1993, 1997). After repeated pairing with reward, the previously neutral sensory stimuli become “reward-predicting cues” and cause dopamine release, while over the same period, dopamine release in response to the actual reward decreases. We refer to this as “transfer” of the dopamine response from reward to reward-predicting cues (see Fig. 1a).

### 1.2 Actions of dopamine at the behavioral level

Without knowing what its effects are, knowledge about the release of dopamine and its transfer from rewards to cues has little explanatory power. Several distinguishable, but not mutually exclusive, hypotheses have been proposed for the actions of dopamine, namely: positive reinforcement, reward, hedonia, incentive motivation, and conditioned reinforcement (Wise, 2004).

The most established hypothesis is that dopamine mediates positive reinforcement, which by definition increases the frequency of responses that it follows. Depleting dopamine or blocking its action reduces the reinforcing effects of food, water, or direct stimulation of dopamine cells (Beninger & Freedman, 1982; Lynch & Wise, 1985; Wise, 2006). Under such conditions, learning new responses is prevented, previously trained responses decline in frequency, and acquisition of Pavlovian stimulus-reward associations is reduced (Darvas, Wunsch, Gibbs, & Palmiter, 2014). Conversely, stimulating the dopamine-rich areas of the brain (ventral tegmental area and substantia nigra pars compacta of the midbrain and their projections to the nucleus accumbens and dorsal striatum) is sufficient to cause positive reinforcement (Corbett & Wise, 1980; Kim et al., 2012; Wise & Bozarth, 1984). Thus the dopamine neurons of the midbrain are both necessary and sufficient for positive reinforcement of responses.

The positive reinforcement hypothesis of dopamine action suggests that dopamine acts on the memory traces of past responses to increase their frequency in the future. The reward hypothesis, on the other hand, refers to proactive effects of dopamine on future actions. “Reward” in this context refers to stimuli that evoke approach to the stimulus. Dopamine release energizes future responses and approaches to behavior (Gallistel, Stellar, & Bubis, 1974). Dopamine is not necessary for producing these responses but increases their vigor or reduces their latency (Wise, 2004).

A third effect of dopamine is related to incentive motivation. This effect refers to the way sensory stimuli can acquire incentive properties or value by prior association with reward (Berridge, 2000). Sensory stimuli that have developed incentive properties can powerfully control behavior (Bindra, 1974). Such stimuli elicit orientation and approach toward them. They can also produce positive reinforcement in the absence of actual reward, in which case they are considered conditioned reinforcers. The transfer of dopamine release from the time of actual reward to the time of the reward-predicting cue may be what gives these sensory stimuli incentive value.

Another theory of dopamine function concerns its role in pleasure. The dopamine hedonia theory is less well-supported. Although rewards are a source of pleasure, elevations of brain dopamine are not strongly correlated with subjective pleasure (Berridge, 2000, 2007). On the other hand, decreased dopamine release in the striatum in response to rewards is associated with reduced ability to experience pleasure (anhedonia) in depression (Belujon & Grace, 2017; Phillips et al., 2023) and there is evidence that dopamine receptors are important in the action of antidepressants (Willner, Hale, & Argyropoulos, 2005).

These theories concerning the action of dopamine are at the level of behavior and subjective experience. However, it is also important to consider the underlying neural mechanisms by which dopamine produces these effects and the requirements for activation of those mechanisms. In broad anatomical terms, the dopamine neurons of the midbrain project to cortical and subcortical areas, most densely to the dorsal striatum, and to a lesser extent, the ventral striatum of the basal ganglia (Swanson, 1982). At the cellular level, dopamine neurons make synaptic contacts on the same neurons and at the same location as the excitatory inputs to those regions (Smith, Bennett, Bolam, Parent, & Sadikot, 1994) indicating that dopamine acts as a modulator of other synaptic inputs.

### 1.3 Actions of dopamine at the cellular level

Many pieces of evidence indicate that the positive reinforcement effects of dopamine are mediated by strengthening of synaptic connections between the cerebral cortex and the striatum. Dopamine facilitates long-term potentiation of these corticostriatal synaptic connections under certain conditions (Calabresi, Picconi, Tozzi, & Di Filippo, 2007; Centonze, Picconi, Gubellini, Bernardi, & Calabresi, 2001; Pawlak & Kerr, 2008; Reynolds et al., 2001; Shen, Flajolet, Greengard, & Surmeier, 2008; Shindou et al., 2019; Wickens, 2008; Wickens, Begg, & Arbuthnott, 1996; Yagishita et al., 2014). At the cellular level, a three-term contingency has been demonstrated, which requires activation of excitatory synaptic input from the cortex, firing of the postsynaptic striatal neuron, and release of dopamine. This conjunction of activity related to sensory inputs, action, and dopamine results in long-lasting strengthening of corticostriatal synaptic connections (Wickens et al., 1996). Behaviorally reinforcing electrical stimulation of the brain also causes dopamine-dependent strengthening of corticostriatal synaptic connections (Reynolds et al., 2001).

### 1.4 Relating the synaptic eligibility trace and the behavioral delay of reinforcement gradient

Electrophysiological experiments have shown that at the cellular level, there is a narrow time window of a few seconds during which dopamine is effective at strengthening synaptic connections (Cassenaer & Laurent, 2012; He et al., 2015; Shindou et al., 2019; Yagishita et al., 2014). If dopamine is released a few seconds too early, or too late, it is ineffective in promoting strengthening of neural connections. We refer to this time window as a synaptic eligibility trace.

At the behavioral level, there is also a restricted time window during which positive reinforcement can affect response learning (Renner, 1964; Tarpy & Sawabini, 1974). Reinforcement is more effective at shorter delays than at longer delays, and a delay reduces the effect of positive reinforcement on learning (Critchfield & Lattal, 1993; Dickinson, Watt, & Griffiths, 1992; Lattal & Gleeson, 1990). This relation between delay and effectiveness of reinforcement is known as the delay of reinforcement gradient (Tarpy & Sawabini, 1974). With natural rewards and no additional cues, the delay of reinforcement gradient has a half-life on the order of tens of seconds (Perin, 1943).

In contrast to the delay of reinforcement gradient, the synaptic eligibility trace has a much shorter time course of a few seconds (Shindou et al., 2019). However, under conditions of delayed reinforcement, a sensory cue that reliably precedes reward can facilitate learning by anticipatory release of dopamine and thus reduce the effects of the delay of actual reward (Renner, 1964; Tarpy & Sawabini, 1974). Even when there is not an obvious sensory cue, the organism’s response, or the delay itself, can act as a predictive cue (Ferster, 1953; Garrud, Goodall, & Mackintosh, 1981; Winstanley, Theobald, Cardinal, & Robbins, 2004). The predictive cue “bridges” the delay (Cardinal, Winstanley, Robbins, & Everitt, 2004; Grice, 1948) and, thus, there is timely release of dopamine at the cellular level. If this bridging mechanism is disabled by removing all predictive cue signals, for example, by using direct stimulation of brain dopamine neurons as the reinforcer, the effects of reinforcement are reduced by delays of as little as one second (Black, Belluzzi, & Stein, 1985). Thus, it seems that the critical timing requirement for dopamine-dependent plasticity at the cellular level can be met, even when there is a more prolonged delay of reinforcement at the behavioral level, by the transfer of dopamine release from the reward to the reward-predicting cues.

### 1.5 The dopamine transfer deficit hypothesis

We have suggested above that transfer of the dopamine signal from reward to reward-predicting cue ensures that dopamine release occurs at the right time to strengthen synaptic connections at the cellular level, even when the behavioral reinforcer is delayed. However, the success of this depends on the ability to learn the cue-reward association and complete the transfer of the dopamine signal to the cue. We have previously considered the possible consequences of failure to learn the cue-reward association. We refer to this as the dopamine transfer deficit (DTD) hypothesis (Tripp & Wickens, 2008, 2009), as illustrated in Fig. 1b. Other authors have also proposed that reduced dopamine functioning causes altered processing of reward in individuals with ADHD (Levy, 1991; Sagvolden, Johansen, Aase, & Russell, 2005) in ADHD. However, DTD is unique in focusing specifically on the timing of the phasic dopamine response.

In developing the DTD hypothesis, we tried to predict the behavioral characteristics that would result from deficient transfer of the dopamine response from rewards to cues. In a condition where dopamine release did not develop in response to a reward-predicting cue, the dopamine signal at the cellular level would be delayed until the actual reward occurred. Under such conditions, dopamine-dependent strengthening of connections would be reduced or not occur at all. Several predictions follow:

### 1.6 Delay of reinforcement gradient

In the absence of dopamine release by reward-predicting cues, even very short delays of a few seconds would reduce the reinforcing effect of the delayed reinforcer. Thus, deficient transfer of the dopamine response from rewards to cues is predicted to cause a steeper delay of reinforcement gradient.

### 1.7 Partial reinforcement effects

Under conditions of partial reinforcement – a schedule in which not every response is reinforced – dopamine release by cues that are present on every trial would normally provide continuous reinforcement at the cellular level. In the absence of such dopamine release, learning under partial reinforcement will be slower. Furthermore, it is well established that although acquisition of learning is slower under partial reinforcement, the learning that is acquired is more resistant to extinction. This is called the partial reinforcement extinction effect (Myers, 1960). The DTD hypothesis predicts slower learning under partial reinforcement and faster extinction (less behavioral persistence) of learned behavior because of reduced dopamine response to reward-predicting cues when reinforcement is stopped (Tripp & Wickens, 2008).

### 1.8 Integration of reinforcement history

Behavior is not only controlled by individual instances of reinforcement but also by an internal representation of the integrated reinforcement history (Killeen & Sitomer, 2003; Okouchi & Lattal, 2006), so that responses that typically result in reinforcement are selected over those that most recently resulted in reinforcement (Tripp & Alsop, 1999). Failure of dopamine transfer would cause increased sensitivity to individual instances of reinforcement.

In light of these predictions in the following sections, we review behavioral studies on the spontaneously hypertensive rat (SHR) model for ADHD, and humans with ADHD, before considering the implications of variations in dopamine transfer for symptoms and personality dimensions of individuals with ADHD and typically developing individuals.

## 2. SHR behavior

Rodents with genetically determined behavioral characteristics provide opportunities for experimental study of brain mechanisms underlying those behavioral characteristics. Moreover, although they are also complex organisms, experimental animals can be bred selectively to express specific behavioral traits. Inbred strains provide homogeneity of genetic makeup, and cross-breeding can be used to determine if characteristics are genetically linked. Animal models also provide otherwise unattainable invasive and repeated measurements important for identifying underlying neural mechanisms. These have been particularly successful in the neurobiological investigation of mechanisms for positive reinforcement.

Here we focus on the SHR, a transgenic strain with, among other characteristics, an altered sensitivity to delay of reinforcement (Johansen, Killeen, & Sagvolden, 2007; Orduna, 2015; Sutherland et al., 2009; Wickens, Hyland, & Tripp, 2011). The SHR was originally developed as a genetic animal model for hypertension (Okamoto & Aoki, 1963). During selective breeding for hypertension, by chance, some distinct behavioral characteristics became fixed in the SHR genome (Hendley, Atwater, Myers, & Whitehorn, 1983; McCarty & Kopin, 1979; Sagvolden, Hendley, & Knardahl, 1992; Wultz & Sagvolden, 1992). These behavioral characteristics include altered responses to reinforcement (Hill, Herbst, & Sanabria, 2012; Johansen et al., 2007; Sagvolden, 2000; Sagvolden, Metzger et al., 1992), impulsivity (Adriani, Caprioli, Granstrem, Carli, & Laviola, 2003; Aparicio, Hennigan, Mulligan, & Alonso-Alvarez, 2019; Bizot et al., 2007; Fox, Hand, & Reilly, 2008; Gonzalez-Barriga & Orduna, 2022; Sagvolden, Russell, Aase, Johansen, & Farshbaf, 2005; Sanabria & Killeen, 2008), and inattention (Aparicio et al., 2019; Sagvolden, 2000; Sagvolden, Metzger et al., 1992; Sagvolden, Pettersen, & Larsen, 1993).

Consistent with DTD hypothesis, the SHR displays a higher sensitivity to delay of reinforcement than comparison strains (Johansen et al., 2007; Johansen, Sagvolden, & Kvande, 2005; Sagvolden, 2000; Sagvolden, Metzger et al., 1992) and a stronger preference for immediate over-delayed reward (Fox et al., 2008; Hand, Fox, & Reilly, 2006; Sutherland et al., 2009). This higher sensitivity to delay of reward in the SHR, relative to comparison strains, may be due to underlying differences in dopamine transfer to reward-predicting cues.

In other rat strains, there is also evidence that individual differences in responses to rewards and reward-predicting cues are associated with different patterns of behavior. For example, animals display differences in their tendency to approach and interact with reward-predicting cues. Approaching and interacting with the reward-predicting cue is called sign-tracking (Davey & Cleland, 1982), while approaching the location of the reward itself when the cue appears is called goal-tracking (Boakes, 1977).

Dopamine responses to reward-predicting cues and reward locations have been measured in Sprague-Dawley rats selectively bred for sign-tracking or goal-tracking behavior. These measures have shown that animals with a lower phasic dopamine response to reward-predicting cues and higher phasic dopamine response to reward delivery display more goal-tracking behavior, and conversely, animals with a higher striatal dopamine response to reward-predicting cues and lower dopamine response to reward delivery display more sign-tracking behavior (Flagel et al., 2012). Since SHRs are more likely to perform like goal-trackers (Silic, Aggarwal, Liyanagama, Tripp, & Wickens, 2023), their dopamine response to reward-predicting cues is of particular relevance.

Several differences in dopamine function have been reported in the SHR. They include lower basal dopamine levels (Fujita et al., 2003), decreased release of dopamine, and faster time course of dopamine clearance after release (Miller et al., 2012). This faster clearance in the SHR may be due to elevated dopamine transporter expression (Roessner et al., 2010; Watanabe et al., 1997). We have recently reported differences in phasic dopamine release in response to reward and reward-predicting cues in Sprague-Dawley (SD) and SHR strains using fast-scan cyclic voltammetry during a simple classical conditioning paradigm (Li, Huang, Chen, Hyland, & Wickens, Submitted). In these experiments, a previously neutral sensory cue was paired with rewarding electrical stimulation of dopamine cells. We found less phasic dopamine release in response to electrical stimulation of dopamine cells in the SHR than in the SD rats. Further, the SHR showed less transfer of the phasic dopamine response from reward to cues over successive trials. These findings indicate altered dopaminergic dynamics in the SHR compared to the SD, which might contribute to differences in their behavioral response to cues and rewards. However, further work is needed for a better understanding of phasic dopamine signaling in the SHR.

## 3. Translation from rodent model to human personality and ADHD

### 3.1 Steeper delay of reinforcement gradient in rodent models and ADHD

Evaluating an animal model for ADHD requires careful attention to the symptoms of the human disorder. One way to ensure a connection between non-human animal and human studies is to use the same task for both. In some cases, nearly identical tasks have been used in studies of SHRs and humans with ADHD. These include a rat task adapted for use in children, and conversely, a human task adapted for use in rats.

An example of a rat task adapted for children is a fixed-interval (FI) schedule that has been extensively used in the investigation of behavioral characteristics of the SHR (Sagvolden et al., 1993). In that schedule, rewards are delivered for the first response emitted after a fixed period of time has elapsed, but not for responses emitted earlier. The FI is then restarted for a number of repetitions. In such schedules, there is usually a decrease in responses after the reward, followed by an increase in responses toward the end of the FI.

**Figure 2.**
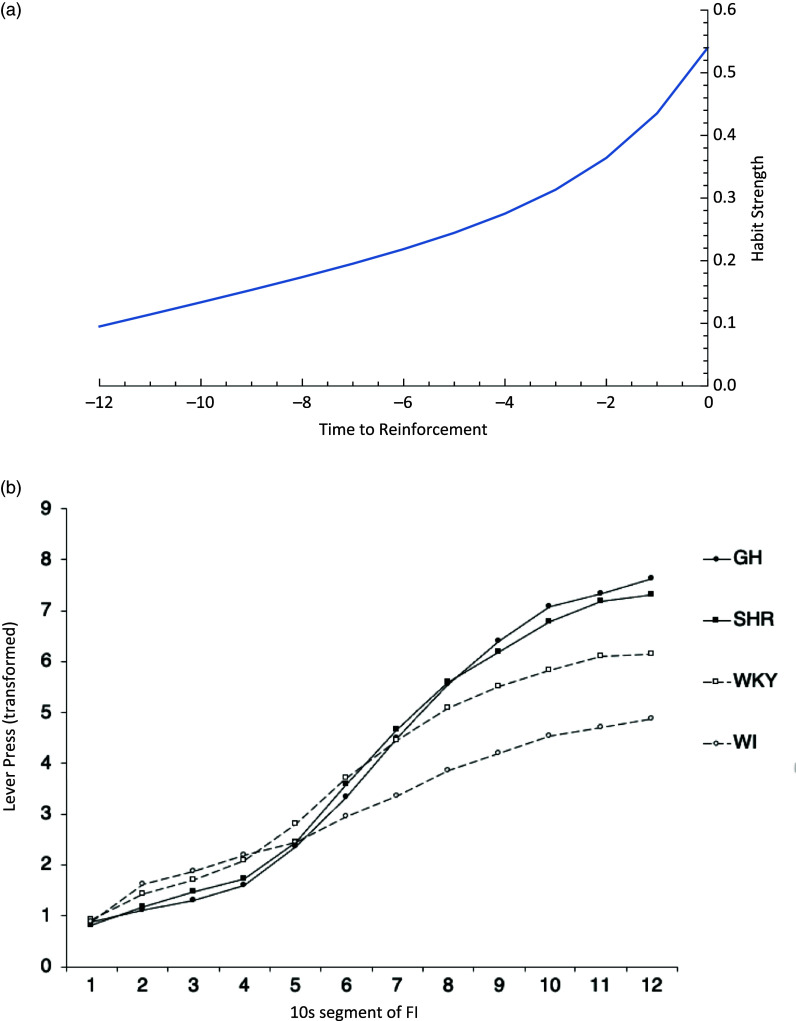
Delay of reinforcement gradient interpretation of fixed-interval responding. (a) Classic representation of the control exerted by delayed reinforcers. Habit strength is plotted as a function of time of reinforcement, based on the formula 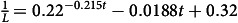 fitted to experimentally measured latency or responses, *L*, for reinforcement at different delay times, *t*, (Perin, 1943). (b) Example of FI responding showing the increase in response rate as the time of reinforcer delivery approaches for four different strains of rat. GH, Genetically Hypertensive, SHR, Spontaneously Hypertensive, WKY, Wistar Kiyoto, WI, Wistar. Redrawn from Wickens et al. (2004).

The characteristic pattern of FI responding has been interpreted as a reflection of the delay of reinforcement gradient. The basis for this interpretation is that a reinforcer not only increases the probability of the response that produced it but to a lesser extent also increases the probability of earlier responses (Catania, 1971; Johansen et al., 2009; Killeen, 2011). This is because, in the FI schedule, the earlier responses in the interval are separated from the reinforcer delivery by a longer delay than the responses occurring later. Assuming that the effects of reinforcement are distributed according to a delay of reinforcement gradient, the earlier responses will be strengthened less than the later responses. Thus, after learning, the earlier responses occur at a lower rate than the later responses (Catania, Sagvolden, & Keller, 1988; Wearden & Lejeune, 2006). An example is shown in Fig. 2b. The increase in responding over the FI is, therefore, treated as an indirect measure of the delay of reinforcement gradient.

On the FI schedule, SHRs produce more responses than comparison strains, with responses increasing more steeply over the FI (Sagvolden, Hendley et al., 1992; Sagvolden et al., 1993). This finding is robust and has been replicated in other laboratories (Orduna, 2015; Wickens, Macfarlane, Booker, & McNaughton, 2004). Thus, their FI behavior indicates that the SHRs have a greater sensitivity to delay of reinforcement than comparison strains.

On a similar FI schedule, children with ADHD made significantly more responses during the FI than typically developing children, with increased responses toward the end of the FI (Sagvolden et al., 1998). As in the SHR, this was interpreted as indication of a steeper-than-normal delay of reinforcement gradient in children with ADHD, operating over a similar timescale of seconds (Sagvolden et al., 1998).

### 3.2 Increased preference for immediate reinforcement in rodent models and ADHD

The converse example of a human task that was subsequently adapted for rats is a signal-detection task developed for studying sensitivity to delay of reinforcement in children with ADHD (Tripp & Alsop, 2001). In this task, children had to press one of two buttons to identify one of two similar stimuli. Correct identification of one stimulus was immediately reinforced, followed by a 3.5 s delay before the start of the next trial. The other stimulus was reinforced after a 3.5 s delay. Both typically developing children and children with ADHD showed a bias toward immediate reinforcement, but the bias in the ADHD group was greater than in controls. These results showed that in this task, children with ADHD are not more sensitive to delay itself, as suggested by the delay aversion hypothesis (Sonuga-Barke, Taylor, Sembi, & Smith, 1992), but specifically have higher sensitivity to delay of reinforcement.

In the animal version of the task, rats were rewarded for lever pressing one of two available levers on each trial (Sutherland et al., 2009). One lever was associated with immediate delivery of a food pellet, and the other lever with delivery of a food pellet after a 10-s delay. Compared to their control strain, SHRs showed a greater bias toward immediate reinforcement. These results show that, like children with ADHD, SHRs have higher sensitivity to delay of reinforcement than comparison strains.

Surprisingly, genetically hypertensive (GH) rats showed similarly greater bias toward immediate reinforcement than the comparison strain. The GH rat was derived independently of the SHR by a similar process of selective breeding for high blood pressure (Simpson et al., 1973; Smirk & Hall, 1958). Like the SHR (Hendley et al., 1983), the genetically determined behavioral characteristics of the GH rat are dissociated from high blood pressure (Wickens et al., 2004). Nonetheless, the GH rat shows FI responding (Wickens et al., 2004) and sensitivity to delay of reinforcement (Sutherland et al., 2009) similar to the SHR. In addition, the GH rat also showed a tendency to be more influenced by individual instances of reinforcement (Sutherland et al., 2009).

### 3.3 Steeper delay discounting in rodent models and ADHD

Individuals with ADHD tend to choose small immediate rewards over larger delayed rewards in both choice delay and temporal discounting paradigms (Antrop et al., 2006; Firestone & Douglas, 1975; Hoerger & Mace, 2006; Jackson & MacKillop, 2016; Marx, Hacker, Yu, Cortese, & Sonuga-Barke, 2021; Patros et al., 2016; Scheres et al., 2006; Sonuga-Barke et al., 1992). Although this is also true of typically developing individuals, the tendency is greater in individuals with ADHD. Most human studies have used temporal discounting tasks, which are also known as intertemporal choice or delay discounting tasks (Marx et al., 2021; Sonuga-Barke, Sergeant, Nigg, & Willcutt, 2008; Sonuga-Barke et al., 1992). These tasks measure the tendency to place less value on rewards that are delayed, often modeled as a discount curve indicating the subjective value of a reward as a function of delay to its receipt.

Although often lumped together with delay of reinforcement, there are fundamental differences in delay of reinforcement and measures of temporal discounting, both in concept and in task design. In delay of reinforcement tasks, the subject (whether human or animal) receives a reinforcer after a delay and the effect of the delay on future responses is measured. In contrast, rewards and delays in human temporal discounting tasks are usually hypotheticals and communicated verbally: they have never and never will be experienced by the individual (Killeen, 2011). Presumably, the decision to delay discounting tasks is based on experience of actual delay of reinforcement in the past, combined with a prediction of how the hypothetical reward will feel in the future after an imagined delay. For example, in such tasks, the participant is given a series of choices between small immediate and larger delayed rewards (such as $50 now or $100 in a year). In the rodent tasks, rats make choices among food rewards that they have been trained to associate with different delays.

Comparison of delay discounting measures between rat strains has yielded equivocal results. Some researchers have reported steeper discounting rates in the SHRs (Aparicio et al., 2019; Bizot et al., 2007; Carbajal et al., 2023; Fox et al., 2008; Orduna, 2015). Others have reported no difference between SHR and comparison strains (Adriani et al., 2003; Garcia & Kirkpatrick, 2013; Gonzalez-Barriga & Orduna, 2022; Ramos, Lopez-Tolsa, Sjoberg, & Pellon, 2019).

It is unlikely that rodents decide based on rather than imagined and discounted future reward, raising questions about whether the rodent tasks measure the same constructs as human tasks (Hayden, 2016). The responses of rodents (and possibly humans) may, for example, indicate the effect of conditioned reinforcers established during past experiences of delayed outcomes (Killeen, 2011; Smith, Southern, & Kirkpatrick, 2023). Humans and rodents show similarly shaped discounting curves, but the discounting rate in humans is several orders of magnitude slower (months) than that measured in animals (seconds) (Hayden, 2016). Nevertheless, the study of delay discounting in animals has provided useful information about the neural substrates involved (Fobbs & Mizumori, 2017).

### 3.4 DTD and symptoms of ADHD

The core behavioral differences predicted by the DTD hypothesis are described in Section 1. They include a steeper delay of reinforcement gradient, slower learning under partial reinforcement, faster extinction of learned behavior, a reduced partial reinforcement extinction effect (as recently demonstrated; Hulsbosch et al., 2023), and increased sensitivity to individual instances of reinforcement. From these core behavioral differences – which are also present in the SHR – we have previously proposed that DTD would cause impulsivity and inattention symptoms described in the diagnostic criteria for ADHD in DSM and ICD systems (Tripp & Wickens, 2008).

The symptoms predicted by the DTD hypothesis include the following: Greater control of behavior by immediate rather than delayed reinforcement would lead to less on-task behavior in situations of infrequent reinforcement. In the absence of constant supervision, unscheduled reinforcing events would control behavior. For example, a child in the classroom may find more reinforcement in looking out the window than attending to schoolwork, for which reward is delayed and infrequent. This would produce symptoms of inattention such as failing to finish tasks and reluctance to engage in tasks requiring sustained effort in the absence of reinforcement. In situations of delayed reinforcement, DTD would also lead to impulsive behaviors such as difficulty awaiting turns or intruding on others because of the immediate reinforcement of such actions.

### 3.5 Personality dimensions relevant to the DTD hypothesis

As clinicians and neurobiologists, we are inclined to view personality as an effect of psychological processes on behavior, based on underlying neurobiological mechanisms. In this view, the correlational structure detected in personality research may reflect the “common cause” of variations in underlying neural mechanisms. However, the empirical basis for the structure of most personality models is self-reporting and analysis of correlations among items, rather than behavioral tests or putative neural mechanisms. Although personality traits can be said to “predict” behavioral characteristics in a statistical sense, such correlations do not establish the direction of causality. Here we suggest that variations in the transfer of phasic dopamine signals from reward to predictive cues, through its effect on core processes related to timing of behavioral reinforcement, is a potential driver of dimensions that are missing or understated in current personality models.

If ADHD is viewed as an exaggeration of normal personality traits, lying at different points on the same continua as typically developing individuals (the “spectrum” model), then we might expect the relevant personality dimensions and ADHD symptoms to share a common neurobiological basis. However, a meta-analysis examining personality dimensions of the Five-Factor Model (which defines five dimensions: neuroticism, agreeableness, conscientiousness, extraversion and openness) in relation to ADHD symptoms concluded that although there was some support for the spectrum model, “the shared variance for all significant relations between personality and ADHD was never more than 50% suggesting that the spectrum model alone does not provide sufficient explanation for the association between personality and ADHD” (Gomez & Corr, 2014). We would argue that this may indicate that one or more relevant dimensions are missing from the personality model. The DTD hypothesis suggests some facets of those dimensions.

Although the DTD hypothesis (Tripp & Wickens, 2008) was originally developed from considering the implications of a putative “deficiency” of transfer of dopamine responses from reward to reward predictive cues, the degree of transfer is likely to vary between individuals. Such variations in association of cues with reward would lead to corresponding variations in specific behavioral characteristics. For example, variation in dopamine response to reward-predicting cues would affect the degree of perseverance in pursuit of goals in the absence of continuous reinforcement. Similarly, variation in the integration of reinforcement history would lead to a tendency to be distracted by individual instances of reward. Also, variations in the delay of reinforcement gradient would lead to different degrees of impulsive choice. Thus, the DTD hypothesis predicts variations in impulsivity, distractibility, and persistence, according to individual differences in the degree of dopamine transfer. These behavioral characteristics do not exactly align with the personality dimensions derived from the Five-Factor model, although there is some overlap, as we discuss in the following section.

### 3.6 Impulsivity, delay of reinforcement gradients, and temporal discounting

Impulsivity is itself a broad concept that includes multiple dimensions. For example, Whiteside and Lynam (2003) proposed that impulsivity includes sensation seeking, lack of premeditation, lack of perseverance, negative urgency, and positive urgency. Alternatively, MacKillop et al. (2016) suggest that the latent structure among multiple measures of impulsivity has three broad categories, namely impulsive choice; impulsive action; and impulsive personality traits, reflecting self-reported attributions of self-regulatory capacity. Such self-report scales focus more on the tendency to act without forethought and inability to inhibit responses, rather sensitivity to delay of reward. However, self-report measures of impulsivity personality traits have weak relations to delay discounting (Bobova, Finn, Rickert, & Lucas, 2009; Janis & Nock, 2009; Odum, 2011a). Therefore, we suggest that the facets of impulsivity, most relevant to the DTD hypothesis, are impulsive choice and lack of perseverance.

Initial studies of the relationship between delay discounting and personality suggested that delay discounting was related to neuroticism and conscientiousness (Mahalingam, Stillwell, Kosinski, Rust, & Kogan, 2014; Manning et al., 2014). However, in a large sample, Yeh et al. (2021) found that discounting was not correlated with neuroticism or conscientiousness scales, and suggested that delay discounting is an important individual difference characteristic in its own right. Thus, the delay discounting rate may be related to an additional personality dimension that is not included In the Big Five, namely impulsivity (Odum, 2011b). Consistent with this, delay discounting measures are proving useful in understanding links between neural systems and behavior in healthy individuals as well as understanding psychopathology (Lempert, Steinglass, Pinto, Kable, & Simpson, 2019).

### 3.7 Persistence

Resistance to extinction – in other words, persistent effort in the absence of reinforcement – is likely to depend on dopamine transfer. As mentioned above, the DTD hypothesis predicted a reduced partial reinforcement extinction effect in children with ADHD, which was recently supported by an experimental study (Hulsbosch et al., 2023). In a meta-analytic review of ADHD and personality measures, inattention was associated with a lack of perseverance (Gomez, Stavropoulos, Watson, Brown, & Chen, 2022), and the association between ADHD and perseverance was moderated by age (stronger in children than adults) and source (stronger in clinical samples than community samples). Thus, persistence appears likely to be a facet of personality dimensions related to variations in dopamine transfer.

### 3.8 Other personality dimensions

There are some suggestions that extraversion may be related to functional variation in dopamine responses that attach incentive value to reward-predicting cues. For example, Depue and Collins (1999) argue that variation in encoding of incentive salience – the intensity of stimulus representations that have become associated with reward through experience – is the main source of individual differences in extraversion. Consistent with this hypothesis studies measuring reward prediction (Smillie, Cooper, & Pickering, 2011) or reward sensitivity (Blain, Sassenberg, Xi, Zhao, & DeYoung, 2021) have shown positive associations with measures of extraversion. More extraverted individuals show a greater preference for immediate rewards (Hirsh, Morisano, & Peterson, 2008; Ostaszewski, 1996). However, although an association of ADHD with higher extraversion has been found in some studies (Gomez & Corr, 2014), the finding is inconsistent across studies and the association may be limited to self-reports (Nigg et al., 2002).

In relation to the remaining personality dimensions, Gomez and Corr (2014) concluded, on the basis of a meta-analysis of 40 data sets, that ADHD symptoms of inattention and hyperactivity/impulsivity were associated with measures of conscientiousness, agreeableness, and neuroticism. Consistent findings have been reported from more recent analyses (Jacobsson, Hopwood, Soderpalm, & Nilsson, 2021). There is no obvious relationship between these personality dimensions and variations in dopamine transfer, although conscientiousness might tap into persistence and agreeableness into distractibility.

## 4. Conclusion

Animal research into the neural mechanisms of reward-related learning has shown that phasic dopamine release associated with unexpected rewards will transfer to cues that predict rewards. This process can imbue cues with incentive value. Sensory cues with incentive value have advantageous effects such as sustaining on-task behavior when reinforcement is delayed, infrequent, or discontinued. Failure to develop such cue-reward associations can lead to a preference for immediacy, increased sensitivity to delay of reinforcement and individual instances of reinforcement, and more rapid extinction of responses in the absence of continued reinforcement. The SHR has provided a useful genetic model enabling investigation of the neural mechanisms underlying these behavioral characteristics. Largely inspired by findings from animal research, we proposed the DTD hypothesis. Several predictions of this hypothesis have been tested and confirmed in both SHRs and humans. Here we further suggest that the behavioral characteristics predicted by variations in dopamine transfer underlie certain dimensions of human personality that may not feature in current personality models. These include variations in impulsivity and persistence. We hope that the insights from rodent models will encourage future studies combining behavioral measures of altered reinforcement sensitivity with symptom and personality measures in individuals with ADHD and typically developing individuals. Such studies may lead to refinement of personality dimensions by inclusion of neurobiological variations in reinforcement mechanisms.
